# Autobiography of the Insane
*"Scenes from the Life of a Sufferer: being the Narrative of a Residence in Morningside Asylum."


**Published:** 1855-07-01

**Authors:** 


### Art. III.—AUTOBIOGRAPHY OF THE INSANE.*

We cannot conceive anything more deeply interesting to the practical physician, so touchingly affecting to the philanthropist, or instructive to the speculative metaphysician and medical psychologist, than the account given by those who have recovered from attacks of insanity, of the workings of the mind, and state of their feelings and sensations, during the existence of mental derangement. We have, in previous numbers of this journal, placed upon record facts bearing upon the subject; and we purpose again directing the attention of our readers to a deeply interesting narrative, illustrative of this section of psychological literature. It appears that the author of the narrative before us published, in the beginning of 1851, in a periodical entitled the "Instructor," a short series of papers in which he detailed the history of his first attack of insanity. The pamphlet

* "Scenes from the Life of a Sufferer: being the Narrative of a Residence in Morningside Asylum."

now records the history of a relapse, &c., which he suffered, and for the treatment of which he was confined in the Morningside Asylum, near Edinburgh.

He says, at the commencement of his narrative, that he purposes "giving a sketch of the interesting and unique community of that valuable institution, with some details of its history and usefulness; all of which—as being illustrative of nervous and mental disease, from the pen of a non-professional observer, actuated by no official predilections or professional prejudices, but who will, with an honest purpose, 'nothing extenuate, nor set down aught in malice,'—may, I trust, be considered both interesting and useful."

After detailing some facts connected with his past life, he exclaims—

"Let no one make light of this disease of the soul! The unreflecting, in the high springtide of health, when their 'bosom's lord sits lightly on his throne,' or those in the enjoyment of robust animal life, may have no sympathy with the victim of this terrible visitation; but how soon may the strong man of indomitable mental energy be laid prostrate in the dust by the derangement of a little nerve in the network of his brain, and have all his pride and power reduced to the imbecility of childhood! The poor nervous dyspeptic is, like the leper of old, shut out from the social endearments of life. To him—

'The sight of vernal bloom, or summer rose,
Or flocks, or herds, or human face divine,'

has no charms. He has lost his way in the world; and the very affections of love, and home, and childhood, where he was wont to garner all his hopes, are to him either utterly perverted, or steeped in the waters of bitterness. Like Hamlet, 'This brave o'erhanging firmament, this majestical roof fretted with golden fire,' why, it appears no other thing to him than a 'foul and pestilent congregation of vapours.' And so, proud man, 'in action how like an angel! in apprehension how like a god! the beauty of the world! the paragon of animals!' this rare quintessence of dust, when a screw gets loose in the complicated idiosyncrasy, becomes a poor craven thing, a moral coward, and the most helpless and pitiable of organized creatures. Such was my own case; and this moral cowardice was the most distressing feature of my malady. I thought I was the basest and most contemptible being in existence, the abhorred of God and man, and the sure object of eternal reprobation; and, in my misery, self-destruction became a fixed idea in my mind:—'Any way—any way, out of the world!' I met with nothing at home but the most devoted gentleness and attention. My wife was to me a ministering angel in all my sorrows, for which she now suffers by failing health; but I have often sadly reflected on the fate of those cast among the rude and unfeeling, who meet with no sympathy, but rather cruel reproaches, for giving way to imaginary woes. Imaginary! 'A man's spirit may sustain his infirmities, but a wounded spirit who can bear?' Depend upon it, reader, that the nervous sufferer would bear mere physical pain, before which you might shrink, with the spirit of a martyr, and would go to the ends of the earth to shake off the *incubus* that weighs upon his brain, and poisons the very fount of life, with every healthy moral perception. He can no more shake off his chronic nightmare by any effort of his will than you can shake off a headache. And let me entreat every humane reader who may honour these sentences with a perusal, to treat the wailings, and even childish eccentricities, of such a sufferer with the utmost forbearance and gentleness, lest he may unwittingly precipitate the fate of a fellow-creature, the blameless victim of a diseased organism, and often trembling on the verge of suicide and madness. How often have I come home in distraction, weary of the burden of life, and resolved to throw myself from a back-window, a height of five storeys! It is painful to go over this dark retrospect; but, as I think a faithful narrative of my sufferings and delusions, with my subsequent merciful recovery, may serve a useful purpose, I shall venture, with fear and trembling, to proceed."

The author soon afterwards, in his mental agony, attempted suicide, by putting a quantity of pungent snuff called "Taddy" into boiling water, and then swallowing the compound; but this proved ineffectual, and only induced violent hiccuping.

"It is, fortunately," he observes, "for persons in my unhappy situation, difficult to procure the more deadly mineral or acid poisons, but my diseased thoughts now fixed upon laudanum as a last resource. I had read the affecting account of poor Cowper, in his efforts at self-destruction, having procured a half-ounce phial of laudanum, as a deadly dose, and I procured, by pennyworths at a time, in different shops, about three-quarters of an ounce, that the quantity, as I thought, might be effective. But, as night approached, and the terrors of death and the judgment stood in array before me, along with the cruel injury I was about to inflict on my poor family, better thoughts got the ascendency, and the deadly draught was thrown over the window, with a resolution to banish such a fearful purpose for ever from my mind. But the demon of self-destruction was not to be exorcised so easily, and it haunted me with the morbid and fixed purpose of moral insanity. I had been so distracted, that for some days I had been unfit to attend to my duties at the chamberlain's office, and I felt as if hurried by an irresistible impulse and inevitable necessity to consummate my terrible purpose. Accordingly, with thief-like caution, and 'method in my madness,' I procured the like quantity of laudanum by the same means as before, and concealed it, till I should go to bed with my sleeping draught, and 'sleep the sleep that knows no waking.'

"This was on Friday, the 2nd of July, 1852. When I think on it, I cannot account for the moral torpor of my mind, but by the conviction that my brain was overwhelmed with insanity. Pity for my poor wife and children, I seemed to have none; and a sense of my moral responsibility to God, as a free agent, must have been greatly obscured or lost. Greedily I swallowed the deadly draught, and lay down in a stupor of misery, never, as I believed, to open my eyes again on this, to me, world of woe. I think it might be four o'clock on the following morning that I awakened to a dim consciousness of existence, and of what I had done. The walls of my bedroom, as I sat up, seemed to be revolving with a vertical motion, and the furniture and pictures on the wall continued spinning round, till my eyes grew sore and my brain giddy with watching their rotatory evolutions. With the exception of a feeling of stupor and giddiness, I felt well and happy; and I lay the whole of that day and next night in a soporific and delicious dream, between sleeping and waking. On the Sunday I walked with my brother in the fields, was very talkative and merry, and went to church in the afternoon. I kept my own council, however, regarding the laudanum, and in the evening I drank tea with my sister in London-street, without exciting any feeling but surprise and apprehension at my apparent rapid recovery and high spirits. I left London-street alone in the evening, intending to visit the grave of a dear friend, Captain Charles Gray, a true-hearted Scottish poet, in the beautiful cemetery of the Dean; but fortunately I had changed my mind, or had felt unable for the journey, as I found myself in the Meadows, when the sun was going down. and bathing meadow, tower, and tree with a flood of golden light. While enjoying the soft effulgence, I was suddenly struck with a faintness at the heart, and a violent palpitation commenced, as if the wheel at the cistern was hurrying on to a sudden crash. Believing I was instantly dying, from the violent throbbing of my heart and brain, it was with difficulty that I reached a seat, and entreated some persons who observed my distress to let my friends know that I was dying. Here, with a crowd gathering round me, I watched, as if for the last time, the sun descending behind some trees on the horizon, and, convinced I had but a few moments to live, the thought of what I had done overwhelmed me with terror and the certainty of eternal pedition. Recollecting that I had observed some discoloured spots on some parts of my body in the morning—no doubt a healthy effort of nature to throw off from the citadel of life the deleterious drug I had swallowed—the thought rushed on me that mortification had commenced, and further confirmed my dread of speedy dissolution. My friends at length came, and took me home, the palpitation having somewhat abated; but my dream-like recollections of the subsequent events of that night and the following day are but the reminiscences of insanity. Still, as in my former delirium, I was obscurely conscious of a double mental agency, knew every object and person around me; and, as there appeared to be a good deal of whispering and watching going on, I thought I was the victim of a conspiracy to deliver me up to the hands of justice as a flagrant criminal. How I passed the night I cannot tell, for I was unconscious of the sorrow and distraction of my wife; but all next day I talked and sung incessantly; and though I am no singer, and not remarkably gifted with the powers of elocution, my recitations and songs, from the ample stores of my memory, seemed so touching and effective, that I shed tears of emotion and joy at my own exquisite utterances. The exalted egotism of the maniac was fairly in the ascendant; but though elevated in my spirits, I was somewhat conscious, from sad experience of the former fiery ordeal I had gone through, that this bewildering excitement was a premonitory symptom of approaching brain fever, and entire mental alienation. I believed I had ruined my character for ever with my employer; but as I was to put a bold face on my infamy, I had determined to resume my avocations next day, and laugh at the simplicity of the chamberlain who kept such a rascal in his employment. Meantime the whispering and plotting seemed still to be going on, and I had resolved to stand on the defensive, and keep a sharp look-ont, when in the evening I was solicited by my brother and other two relatives to accompany them in a short excursion to the country, in a cab. To this I cheerfully acceded, marvelling much where we were going, or what friend we were to visit. I had scarcely taken my seat, however, when I suspected, from their manner, the covert purpose of the drive, and the truth dawned upon me that they were conveying me to a madhouse. But I felt passive and resigned to my fate, thinking I should find a refuge from disgrace, where the finger of scorn, or the reproaches of cruelty or malice would not disturb my solitude and repose; and I voluntarily gave up to my friends my penknife, believing, in my partial gleam of sanity, that I could not safely be trusted with edge instruments. In a few minutes, accordingly, I found myself an inmate of Morningside Asylum."

The author then proceeds to describe his conduct and feelings soon after his admission to the asylum. His advent naturally excited the curiosity of some of the other patients in the establishment, whom he says:—

"Welcomed me into their community with congratulations and laughter. Some eyed me with curious and critical inquisitiveness, and, like all other little isolated communities, were impatient to know who I was, where I came from, and what had brought me there. I told them I was Peter M'Craw, the tax-gatherer from Leith, so graphically described in poor Robert Gilfillan's song, and that I had been driven demented by ill-usage. But I was assured they were all happy there, there were no taxes to pay, and everybody laughed at the folly of the world without; an assurance which was corroborated by a hearty peal of laughter. After stripping and getting into bed, I continued, notwithstanding the remonstrances of the attendants, to be very noisy; I could not sleep, nor allow others to sleep, and I could not lie still, from nervous excitement; and I was forthwith hurried away to another part of the house, through a long line of corridors and echoing galleries, where I was put into a separate apartment, and locked up, with night, and solitude, and a distempered brain, in a madhouse. I was not yet, however, under the influence of terror, though somewhat confounded by my unceremonious reception; and having some exaggerated notion of my own importance, I believed I was confined through some political manœuvre connected with the pending election of Mr. Macaulay, who had some time before sent me a copy of his "Lays of Ancient Rome," with a complimentary letter on my poetical efforts. Thus the spirit of a martyr for a little sustained me. I thought an acquaintance who had died in the Asylum ten years before was still living, immured in one of its cells, and a bonnet which was lying beside me seemed the identical bonnet that he wore; and I somehow comforted myself with the assurance that my friend, Mr. Combe, would visit me next day, and, penetrating the secrets of my prison-house, would not suffer a person of my importance to be robbed of his liberty. I tried in vain to sleep, but the hardness of my bed, suiting ill with the extreme attenuation of my body, would not suffer me to rest, while the nervous state I was in, and the dreadful noises that now assailed my ears, entirely put to flight nature's soft nurse, and threw me into a horror of great darkness and misery. Being rather sensitive in regard to personal cleanliness, before the daylight had faded, I was shocked to observe stains on the bed, a thing purely accidental and exceptional; while a utensil of gutta percha—(earthenware being obviously inadmissible in such a place)—distressed me with its strong ammoniac odour. In the apartment on my right, a poor maniac raved through a blasphemous form of prayer the whole night, cursing God, as he called it, with all the bitterness of his heart and tongue; while in that on my left another old madman reasoned high on the perplexities of fate and free-will, faith and works, with all the energy of a Calvinistic divine, and never seemed to sleep a wink. Another shouted and sung through the watches of that dreadful night, 'Cain was a murderer! Cain was a murderer!' which ran through my very soul with terror, as a denouncement and reproach levelled at myself; while the swearing and blasphemies which ever and anon startled the dull ear of night, blending with my distempered fancy, threw me into a delirium of insanity, and were enough to whirl the soundest brain. I now thought I was cast into hell, and herding with the damned, beyond all reach of hope or mercy, and my sensations under this delusion were indescribable. Anon 'a change came o'er the spirit of my dream,' and I thought I was in my grave, buried alive, deep, deep in the bowels of the earth. I gasped for breath, and marvelled that there should be life in the tomb, or any sense of its horrors. I pinched my body, and groped about the walls to be assured that I was a living man, and to get out of my perplexity. But these wild hallucinations overwhelmed my tottering reason; yet I never entirely lost consciousness and memory, and can look back on the whole drama like the phantasmagoria of a troubled dream.

"The cold grey dawn of the summer morning at last broke in upon my delirium, but its uncertain light at first gave greater scope to my disordered imagination, which converted the folds of the bedclothes into serpents and reptiles, and all sorts of loathsome creeping things, 'hydras, gorgons, and chimeras dire.' With this impression stamped on my brain, I started to my feet in horror, with my eyes riveted on the hideous sight; and there I stood transfixed, and unable to move for many minutes, in unutterable terror. At length, slowly reaching down my hand, as daylight increased, to touch one of the immovable monsters, I was mightily relieved to find nothing but the folds of the bedclothes, and that I myself was the only living thing in the room. My two next-door neighbours still, at intervals, continued their exercises; and an occasional howl and rhapsody of oaths fell on my ear, and testified that I was still somewhere in the land of the living. But I had now lost all consciousness of where I was. I felt exceedingly unwell and feverish after so much agitation, and would fain have slept, but no slumber would visit my eyelids, and from the increasing commotion I heard, the business of the day seemed to be commencing. By and by my door was opened, and my clothes flung down upon the floor, but no one spoke to me, till a stout, good-natured looking man came in with some coffee and bread, and spoke kindly to me while I took breakfast. I then managed to dress, and walked out into a court, where I felt delighted with the freshness of the morning after the horrors of such a night. Here I saw a most *outré* group of human beings moving about—epileptic, idiot, and fatuous persons, with all the miscellaneous oddities and eccentricities of a madhouse. I did not suspect I was in Bedlam, but imagined I was in some hydropathic establishment in the neighbourhood of Glasgow. I began to feel my brain getting clearer, and reason partially resuming her seat, though I was perplexed to recognise in the persons about me friends and relatives, no doubt arising from some obscure association or resemblance, one of whom was a son of my own, who, poor fellow, was then far away on the deep, deep sea, but none of them could I get to understand or communicate with me, which distressed and puzzled me very much. I was now cheered by a visit from the medical gentlemen, who inquired kindly into my condition, and gave some orders regarding regimen and the bath. I kept in the airing-ground the greater part of the day, but towards evening my hallucinations returned, and, though I was conscious of sitting on a bank opposite a wall of the court, I could not shake off the impression that I was in my own bedroom, and that some one was listening at the keyhole; thus confirming the theory of the dual organization of the brain, which had lost its balance, one section being partially sane, while the other was utterly crazed. To my solitary apartment, and to bed, again I went, but not to sleep. The poor maniac on my right again commenced his revolting blasphemies, and he on my left his controversial monologue, while the same stunning noises and howlings, with 'Cain was a murderer!' again assailed my ears. I got through the night, however, without the aid of 'tired nature's sweet restorer,' with less misery than the preceding, and was glad when I was called in the morning to enjoy the refreshment of the tepid bath. Then the sweet breath of the morning, while 'the opening gowan wet wi' dew' spangled the fragrant grass in the courtyard, went to my heart with its freshness, cooling my fevered brain, and bringing tears of grateful joy to my eyes. But the thought that I was deserted by 'all the world and my wife,' and an object of scorn and abhorrence to my friends, was ever uppermost. One of my greatest privations was the want of snuff, and in the course of the day I was much gratified by the receipt of a parcel containing a supply of that necessary article, with other memorials of kindness, which I knew could only come from the true friend of my home and heart. This was the best medicine to my bruised spirit, and helped to remove my suspicious dread of desertion and contempt. The mental fog was clearing away, and I entered into communication with some of my companions, who seemed very willing to take me under their protection. An arithmetician instructed me in figures, a ' stickit minister' in divinity, and a crazed flunkey, who assured me they were all mad but himself, maintained that Dr. Chalmers was the editor of a certain journal, and that he himself knew Robert Burns (who died long before he was born), and assisted him in the composition of some of his best poems. So 'time and the hour' move through the day, and another almost sleepless night, disturbed by the same demoniac noises as before."

On the morning after his admission, he became sensible as to his exact position, knew that he was

" In the Asylum at Morningside, and felt also satisfied that my friends had done wisely in removing me to the security of such a retreat. I felt that I had been snatched from destruction by the merciful interposition of Providence, and that I would yet be permitted, with renovated health, to resume my place and usefulness in society, and feel the endearments of my own fireside. These sane thoughts came over me with a healthful and exhilarating influence, and I lay down at night in my weary solitude with more comfort than I had felt for many a day, and got some snatches of sleep, notwithstanding the pandemonian noises around, which were now becoming familiar. Through the day I now found rational companionship and literary conversation with an educated man, who had been bred for the church, and who had carried off several prizes for his proficiency in mathematics. But he turned out too rude and self-important for me, his disease evidently being an inflated notion of his own consequence as a gentleman, which, with his boorish manners, would obviously render the unfortunate man unfit for society. But I found here a brother poet of no mean pretensions, and, as a prose writer, a man of unquestionable talent—the author of a volume of very considerable merit. He kept his room during the day, busy with his book or pen, and came out for an hour or two in the evening to walk round the airing-ground. He proved a good talker, and a very interesting companion, was full of anecdote and humour above the common pitch; and, having seen much of the world, and read a good deal, though somewhat loose in his opinions, we went over the world of books and authors together with mutual pleasure, and never, for the short time I enjoyed his society, flagged from a dearth of matter. These evening reunions were further enlivened by an old man, J—— G——, the same who reasoned on divinity all night—who walked before us good-naturedly on our rounds, serenading us on his fiddle with all his might, and occasionally interpolating an observation of his own in his peculiar line of metaphysics. Honest J—— seemed to respect us as philosophers, and we gladly accepted of his homage. My friend was the crack contributor to the 'Morningside Mirror,' a miscellany printed and published in the Asylum, and written by the patients; and his contributions, both in prose and verse, would do no discredit to works of far higher pretensions. Poor C——! his malady seemed to be exalted ideas of his own consequence, and of his great and even royal lineage, combined with unfortunate social propensities, which had crazed his brain, and driven him into a madhouse. He left the Asylum shortly after I knew him, and I should rejoice to learn that his mental health is confirmed, and that he had found useful and salutary occupation for his talents."

The acute symptoms of the author's attack having subsided, he was removed to what he terms the third gallery of the Western Asylum, appropriated to convalescents. He says—

"Here I found myself in a comfortable parlour, among about a score of quiet, rational-looking men, some of whom appeared attentive and polite, and welcomed me into their society with a frank, homely courtesy. After breakfast, and looking over some periodicals and newspapers, with which the patients are here supplied every Sunday morning, I attended for the first time the forenoon service in the chapel, under the pastoral ministration of Mr. Lorimer, the chaplain. Here I found about three hundred patients, with their respective attendants, assembled from all departments of the asylum, and was very much struck with the stillness and propriety of their demeanour, contrasting favourably with the levity and ostentatious parade often exhibited in some more fashionable places of worship. Here were the imbecile and fatuous by nature, with the hopeful convalescent; the confirmed maniac, with the peculiar grey light of insanity glittering in his troubled eye, and the inexplicable monomoniac, with his fixed delusion—rational and intelligent on every point but one; the moping idiot, the brain-struck epileptic and paralytic, the demented victim whose mental life and light had been obscured or extinguished by misfortune, and whom the world had cast forth as lumber; the unhappy victim of nervous hypochondria, with unhinged brain, who meets from the healthy and inconsiderate with more reproaches than pity: here were they all assembled, men and women, young and old, with all their delusions and woes, reverently inclined to join in the worship Him who had seen meet to afflict them with the heaviest of human calamities, and lifting up their voices in cheerful praise to the common Father and Everlasting One, whose mercy endureth for ever. The service was judiciously short and varied, and seemed to have a soothing and beneficial influence; for, amidst all the moral perversion and obscurity of thought among the insane, the one grand idea of God above, and the better land, seems never to be extinguished. Here I got out to a new airing-ground, and a new society, and a new sphere of observation. In the afternoon I had an opportunity of writing a cheerful letter to my wife, who, I learned, had called every visiting day, but whom I had not yet been permitted to see. At night I went to the same bed again in the dormitory, from which I had a week before been so unceremoniously expelled; but during those six nights so agitated and unwell had I been, that I did not sleep as many hours. When the drowsy goddess with leaden sceptre would press on my eyelids, a feeling of horror, a sensation as of impending death, came over me, which made me both to long for and dread the approach of sleep. But I had now sufficient tranquillity to read; and with the grey, growing light of the summer morning, I beguiled many an hour with a book, till the bell at six o'clock tolled the welcome advent of a new day, when, with my strange bed-fellows, I gladly rose, made my bed, and got a *cold* shower-bath, now substituted for the *tepid*, the electric shock of which dispelled all the vapours of hypochondria, and restored me to myself. I was terribly distressed with the extreme emaciation of my body, and sitting on the hard seats gave me much discomfort, as I was unable from weakness to keep long on my feet, or to walk much; but I was encouraged to think that in the land of life and hope I would yet be enabled to conquer all my sorrows. Shut up within the walls of this little world, one day was exactly like another in its monotonous course; but I now had a new world of books in the small library of the Asylum, and a most novel and most interesting world of life in the strange society around me."

Being now transferred to the care of Dr. Rowe, he was requested by that physician to contribute a poetical piece to the "Morningside Mirror;" and accordingly, invoking the aid of the Muses, he, under their inspiration, wrote one day, whilst reclining on a bank in the airing-ground, when "bathed in sorrow and tears," and surrounded by the "babbling and interruptions" of his poor companions in affliction, the following:

INVOCATION TO HOPE.

Star of the crushed and bleeding heart!
Thy mildest influence impart,
To soothe a pilgrim's woe;
Wrecked on the leeward shores of life,
Unequal to the storm and strife,
That all must share below.

Piercing through sorrow's darkest dream,
O let him feel the glorious beam,
That lights the soul to God:
Like day-spring from on high descend,
That he may see the gracious end,
And kiss the chastening rod.

Like clouds of floating incense, roll
Immortal visions on his soul,
That he may feel the glow,
The fragrant amaranthine bloom,
That springs in realms beyond the tomb,
Untouched by human woe.

With introverted eye, no more
The secret springs let him explore
Of his corrupted heart,
But look to Him, the undefiled,
With all the faith that warms a child,
Unchilled by human art.

Love inconceivable and pure,
A righteousness that shall endure,
Will then his thoughts employ:
His sorrows, that no tongue can tell,
Who triumphed over death and hell,
That we might share his joy.

Lead him to fountains fresh and clear,
Where dreams of childhood may endear,
In sweet perennial bloom,
And soothe his sere heart's withering woe,
That wraps all lovely things below
In shadows of the tomb.

O! in the realms of life and hope,
No more in darkness let him grope,
Like wretch without an aim;
But strong of purpose and of will,
The true and faithful part fulfil,
That love and kindred claim.

Ah! what were life, and what were death,
If reft of love, and hope, and faith?
A gulf of dark despair!
But, fired by these, the enraptured soul,
Pierces through time's obscurest goal,
To scenes divinely fair.

Divinely fair! creation young,
When God's own sons in triumph sung,
And hailed the dawn of time;
What shouts of angels and of men,
Will hail Emanuel's glorious reign,
In Heaven's eternal clime.

Give him through mists obscure to trace,
The glories of creative grace,
Of dignity and love,
In nature's face serenely fair,
In all that thrills the vocal air,
Or warbles through the grove.

Thou rising and thou sinking sun,
Bright emblem of the Eternal One,
O light again his eye,
Like patriarch of old descried,
To meditate at eventide
In solitary joy.

Star of the beautiful and true,
Once more descend like evening dew,
Or morning's genial beam ;
With song of lark and breath of balm,
O find or make thy suppliant calm,
And soothe his maniac dream.

O seal his eyes in dreamless sleep,
That he no more may wake to weep,
Starting in horror wild;
Or lap his soul in dreams of youth,
Warm with the glow of love and truth,
That charmed him when a child.

With food and raiment give content,
And all the good by mercy lent,
With grateful heart to prize :
A body sound, and healthful mind,
And hope that's not to earth confined,
But centered in the skies.

After referring to the great amelioration in the treatment of the insane introduced since the days of the celebrated Pinel, the author observes:

"My own youthful recollections of a madhouse were associated with all the horrors of a solitary cell, cruel coercion, the clanking of chains, and the howlings of despair, from having frequently, when a boy, witnessed such scenes in the Bedlam of my native place, one of the earliest public institutions of the kind in Scotland. Ah! could I then have dreamed that I myself should one day be the inmate of an asylum, the terrible conception would surely have whirled my brain, so miserable were the impressions of what I had seen on my youthful mind. But how well it is for us, that

'Heaven in its mercy hides the book of fate,
All but the page prescribed—our present state.'

'Else,' as Pope justly adds, 'who could suffer being here below?' Bedlam was then one of the regular sights of the place, and often a spectacle to gratify the idle and unfeeling curiosity of vulgar minds, which could feel any gratification in looking upon this last of human afflictions—the temporal wreck of an immortal mind. Often have I accompanied the keepers at supper-time, when doling out to the poor creatures their portion of potatoes and salt (but I rather fear the latter condiment was sometimes dispensed with), and I can never forget the wild, startled look of many a cadaverous visage which the grating lock and the unwonted light roused from its wretched lair. To some, chained among straw like wild beasts, their food was thrust through a loop-hole in the wall, their only window, while others were left to devour theirs in the dark as best they might. The more harmless or convalescent patients—if such a condition as convalescence was then recognised in such places—were assembled in the evenings and portions of the day in a common, ill-ventilated room, under the charge of a keeper, armed with a terrible thong (the same with which poor Abban Hassan, of the 'Arabian Nights,' suffered his flagellations), and a supply of straitjackets for the unruly. Frequent were the scourgings with this instrument of torture; and a supplementary infliction was readily found in a pump in the court, surrounded by a box, into which the refractory patients, male or female, were thrust, while a pitiless torrent of water was poured for a long time on their distracted brain. Sunday was a day of unmitigated solitude. No voice of prayer or praise hallowed the day of rest; and the only sound that met the ear of the citizen enjoying a quiet walk in the fields on that blessed day was the shrill whistle of some solitary wretch, or

'Moody madness laughing wild amid severest woe.'

"But I turn from this heart-saddening spectacle, with its many untold tales of unutterable woe, to the cheering atmosphere of life and light, which sheds a spirit of hope and comfort over the beautiful precincts of Morningside Asylum. At ——, the fearful motto of Dante might appropriately have been written on the portal. Here words of hope and consolation might adorn the gateway, speaking better things to the unfortunate and their friends in the day of calamity, than our forefathers ever dreamed of in the dark days that are happily for ever past."

The following description of one of the weekly balls at the asylum will interest our readers:—

"Strangers are always expected, and every one very properly wishes to appear to the best advantage, and to acquit themselves with propriety, in honour of the event. Accordingly, at seven o'clock, from all departments of the asylum the patients, accompanied by their respective attendants, came trooping on the tip-toe of expectation for the ball-room. On entering the spacious and brilliantly-lighted hall, I was never more struck and interested than by the spectacle that met my gaze. Here were from 300 to 400 persons of that class, who were formerly considered beyond the pale of social intercourse, like the lepers of old—pariahs of the human race—assembled for the exhilarating and healthful enjoyment of music and the dance, and forming as decorous and wise-like a festive party as could be found in all broad Scotland. When arranged for the dance—which is gone about with the utmost propriety and politeness, each gentleman courteously selecting his own partner—the *tout ensemble* of this extraordinary and unique spectacle must astonish and delight every stranger. First comes a Scotch reel. Perhaps from forty to fifty couples wait with glistening eye the starting note, when off they go, with 'life and mettle in their heels,' making the walls of the stately mansion vibrate to their vigorous tread, as if sorrow and despair had never followed their footsteps, or cast a shadow over their path. Grotesque and odd enough are some of their motions; and, as the 'mirth and fun grow fast and furious,' to watch their rapid evolutions, as I do with my mind's eye at present, seems like the phantasmagoria of a wizard dream. It does not suggest the idea of Bedlam broke loose, but of Bedlam in ecstasy, till the fiddles give their closing scream of discord, when the whirling group is arrested, and with many a profound bow, and politely leading of partners to their seats, the assemblage is all in an instant quietly seated again, the ladies on one side of the hall, and the gentlemen opposite, while the strangers are set apart on the orchestra side. But now a song is announced by the master of the ceremonies; and anon a voice is heard from among the group of patients, chanting very sweetly Ballantyne's pretty nursery song of 'Castles in the Air,' which is listened to in eloquent silence, and rapturously applauded at the close. I may here be permitted to observe, that on another evening I was secretly gratified by hearing a song of my own, 'My Bosom Flower,' sung by Dr. Rowe, with his fine vocal powers, the author being unknown to all present, and congratulating himself in his obscurity. Quadrilles, country dances, and every variety of exercise for the 'light fantastic toe,' succeed, in which the delighted patients acquit themselves admirably; and so, alternating with the song and the dance, the evening passes away, winged with delight, till between nine and ten o'clock, when the Queen's Anthem, finely and heartily sung by the whole assemblage, closes the extraordinary and gratifying scene."

The author then details the history of his gradual restoration to mental health, of the advantages he derived from the shower-bath, active exercise in the open air, attention to diet, and the regulation of the stomach and bowels. Like many patients in a similar stage of convalescence, he expressed an anxiety to return to his own fireside, under the conviction that the storm had passed away, and the black clouds that had been hovering over him had all dispersed. He says—

"I now felt my health so much improved, that, urged by strong necessity and every motive of duty and affection, I began to look about for some employment; and through the influence of my friend Mr. Ballantyne, and with the sanction and recommendation of Dr. Skae, I got an appointment as a collector for the Edinburgh Water Company. This, however, after a week's trial, I found so much beyond the compass of my strength and faculties, that, with feelings of deep mortification and disappointment, I was forced to relinquish it. I became perplexed with the simplest calculations. I lost money, and I literally lost myself, having, on the last day of my collecting, become so bewildered in streets long familiar, that I could not discriminate north from south, or east from west, as if my brain were completely turned. I became very much alarmed, and went next day to the asylum, to consult Dr. Skae, who immediately made arrangements for my return; and accordingly, on the evening of the day following, I was replaced in my old quarters, and, fortunately, just in time to avert the dreaded relapse of a diseased brain. The cloud of hypochondria was hovering over me, and threatening to wrap my spirit again in its dusky folds; but, as it is only those who have felt the iron of such a malady enter their souls that can sympathize with or understand me, I will not inflict on the reader any further allusion to a nameless misery, that can only find adequate expression in the pathetic and terrible eloquence of the Book of Job."

After his return to the asylum the author was allowed to resume all his former privileges. For some time previous, he was in the daily habit of visiting the billiard-room of the old house, a recent addition to the comforts of the asylum, and a boon of great value to many of the patients. Here he regularly saw the newspapers, and the leading periodicals of the day, and, what was of great importance to his personal comfort, he had the luxury of a sofa, or stuff-bottomed chairs, on which to rest his attenuated frame, a pleasure which was further enhanced by the society of a superior class of patients, of obliging gentlemanly manners. Like the poor lunatic of Crabbe, Sir Eustace Gray,

"They would, with free and easy air,
Appear attentive and polite;
Would veil their woes with manners fair,
And pity with respect excite."

The writer then proceeds to describe some of his companions. He observes:—

"A very beautiful billiard-player was Mr. ——, an old inmate of the house, and quite a psychological curiosity. He seemed like a man walking in a dream; and indeed the strange delusions of lunacy, and more especially in the case of my poor harmless friend, bear a remarkable affinity to the phenomena of dreams. The most absurd and improbable things do not strike the dreamer as being either absurd or improbable, but are stamped upon his brain and his senses with all the force of reality; and while one faculty is in an abnormal state of action, the presiding judgment, or the power of comparison and causation, is totally in abeyance. In our friend's case, historical events and personages, from the dream-land of memory, were perpetually mirrored on his brain, but, like the images in a broken mirror, in disjointed fragments. I was greatly amused by the conversation of the polite old gentleman. The highest compliment he thought he could pay me, was to suppose me four thousand years old; for the events and persons of the present generation were as but of yesterday, and unworthy of notice. A portion of his extraordinary reminiscences may be worth recording, not in the spirit of levity or ridicule, but, as I said, in the light of a psychological curiosity:—

"Oh yes, Mr. ——, I knew old Noah very well! There were two Noahs whom I knew; but old Mr. Noah lived some thousand years before the Noah you refer to, who built the ark. I had a good deal to do with the construction of the ark, and furnished some very useful hints in regard to the admission of light and air, and so forth. He was a very respectable man Noah, with a decent family, but unfortunately he got into very dissipated habits in his old age, and, in spite of all I could say to him, he indulged in brandy and water, to a very hurtful excess!

"Julius Cæsar was a very clever man, with a bald forehead; but I was more intimate with Alexander the Great of Macedonia, as I was long in the military profession myself. I one time commanded three millions of men about three quarters of an inch tall. No; they were not Lilliputians. I knew Captain Gulliver very well. And they were smart enough little fellows, but my men were excellent marksmen—they always aimed at the eyes, and never missed. I'll tell you, Mr. ——, the most extraordinary thing you ever heard, which beats railroads. I was once transported from the farthest shores of India to the centre of Africa in three minutes!' 'By what means?' he repeated in reply to a question respecting his method of transit. 'By a bomb!' In reply to my remark, on the danger of being wafted so rapidly over vast oceans, he continued, 'Yes; it was attended with considerable danger. I once came down souse into the ocean; but fortunately I hailed a vessel, which came to my relief, and I pursued my journey to the wilds of Africa, with the loss of only ten minutes!' Sometimes, however, the poor gentleman would seem doubtful of his own veracity, or the strength of his memory, and remark, 'My memory is not so good as it was, and my health, for the last hundred years, has rather failed me, which makes my head a little confused.' And thus he moves about in his waking dream, wearing out his existence between his pipe and a game at billiards, diversified occasionally by a short excursion in the neigbourhood, in charge of an attendant."

The author, after recording some historical facts connected with the origin and progress of the Asylum, observes, when speaking of some of the causes of insanity,—

"It is melancholy to think, that, of all the causes of insanity, intemperance is found to be the most prolific; a terrible result that may well make the drunkard pause in his infatuated career. Of the cases admitted into the Asylum during 1852, no less than 50 were from this cause alone—34 males and 16 females; the males being usually as two to one of the females in these cases. This amounts to the startling number of 20 per cent. of the whole cases admitted; and, apart from the females, to 26 per cent. of the males.

"But there is an obscure, though very frequent cause of insanity, little known as such, and seldom adverted to, for obvious reasons, though well known to those familiar with the habits of the insane, which I simply allude to.

"A very general delusion I found to be a belief in some mysterious and unseen agency, such as electricity, mesmerism, or spirit intercourse (a prevailing delusion not confined to Bedlam), of which many think they are made the victims by the doctors, or some imps of darkness. One patient is firmly persuaded that he was hunted out of America by this devilish agency, followed by the doctors, who were concealed in the vessel, across the Atlantic, and finally landed in Morningside Asylum, where the same parties still operate on him with their electric experiments. Apart from this fixed delusion, he is perfectly sane, and a very obliging and useful person. Another man is similarly tormented; and believing he is acted upon through the medium of water, has a great horror of that fluid, actually turning pale when he sees any one wash their hands. In the parlour and bedroom of which I was an inmate, I had long observed a very quiet, sensible-looking man, and was curious to know what had brought him there, or how he was affected. For this purpose I sometimes talked with him, but could get no clue to his malady, or the trace of any insane symptom, his only peculiarity being, that he was constantly, when not at work in the grounds, reading his Bible. One day, however, on asking how he did, he solved the mystery, by telling me that he would be quite well, if they would let him alone with their electricity. Another, a most useful and much-esteemed patient, thinks there is an electric machine in his head, caused by the swallowing of ground glass.

"Apart from the large class who are imbecile and idiots by nature, I think, from my own observation, that the bulk of those afflicted by mental disease, are originally of weak, and seldom above average intellect; and that very few could reply as did the celebrated Robert Hall, to the impertinent question of a foolish person, as to what had brought him to a madhouse, 'What will never bring you here—too much brain.' Of all the multitudinous causes of insanity, these fixed delusions are found the most difficult and hopeless to deal with. The brain has somehow got an unaccountable twist, and to attempt to reason with them on their preposterous fancies is quite absurd, as on that particular point they have unfortunately no reason to appeal to, and it only makes them angry; and no doubt the irritation they are subjected to by inconsiderate contradiction when at liberty, renders them unfit for social or domestic life.

"I was always very much struck with the sedative influence which an asylum has on new patients. Though brought there often manacled and stark mad, it seemed as if by instinct, and 'to the manner born,' they fell at once into the routine of the place, and were soothed or subdued by the scene and the atmosphere around them. One poor fellow was brought bound hand and foot, his distracted friends thinking their very lives in danger from his violence. He was instantly released from his bonds, and soon appeared parading the galleries among the other patients, dressed in the ordinary costume, and perfectly harmless, as 'one of us,' though still distinguished by a sullen pride, and a stern but passive resistance to all conciliation or inducements to any sort of work. This in general pleasant result must arise partly from the feeling of protection and security which an asylum affords, and partly from the sense of a power and authority which it would be useless to resist. The sleeping dormitories—containing, as I said, sometimes twenty beds, so clean, well-aired, and comfortable—have also a tranquillizing and excellent effect. In mental disease, 'it is not good for man to be alone.' Night, darkness, and solitude are the parents of phantasy and terror, and more especially with a disturbed brain; but in these dormitories a feeling of society, cheerfulness and light—a jet of gas, with fires in winter, being properly kept burning all night, and an attendant among the sleepers, diffuses great comfort, and dispels the sensations of terror. These good effects, it may be useful to observe, are most strikingly exemplified when the disease is taken in its earliest stage; the chances of cure, as proved by statistical evidence, being then as four to one in favour of the patient."

We cannot afford the space to quote more at length from this interesting *brochure*. It has its faults and exaggerations, but we pass them over, being only anxious to select those portions of the pamphlet that are likely to amuse and instruct our readers. As an episode in the life of a man who has suffered from an attack of insanity, and as giving an insight into the interior of one of the principal Scotch Asylums, we think the work will be useful to the public.

